# Metabolomics Analysis Discovers Estrogen Altering Cell Proliferation *via* the Pentose Phosphate Pathway in Infertility Patient Endometria

**DOI:** 10.3389/fendo.2021.791174

**Published:** 2021-11-15

**Authors:** Yingxin Zheng, Yuemeng Zhu, Ting Zhuge, Bin Li, Chao Gu

**Affiliations:** ^1^ Department of Obstetrics and Gynecology, Obstetrics and Gynecology Hospital of Fudan University, Shanghai, China; ^2^ Shanghai Key Laboratory of Female Reproductive Endocrine Related Diseases, Obstetrics and Gynecology Hospital of Fudan University, Shanghai, China

**Keywords:** metabolomics, estrogen treatment, hysteroscopy, pentose phosphate pathway, 6-aminonicotinamide

## Abstract

Estrogen therapy is widely used as a supplementary treatment after hysteroscopy for female infertility patients owing to its protective function that improves endometrial regeneration and menstruation, inhibits recurrent adhesions, and improves subsequent conception rate. The endometrial protective function of such estrogen administration pre-surgery is still controversial. In the current study, 12 infertility patients were enrolled, who were treated with estrogen before hysteroscopy surgery. Using cutting-edge metabolomic analysis, we observed alterations in the pentose phosphate pathway (PPP) intermediates of the patient’s endometrial tissues. Furthermore, using Ishikawa endometrial cells, we validated our clinical discovery and identified estrogen–ESR–G6PD–PPP axial function, which promotes estrogen-induced cell proliferation.

## Introduction

Infertility is defined as a failure to fall pregnant despite a couple having regular unprotected sexual intercourse for over 1 year (1). Although infertility has multiple causes, regular screening, including semen analysis (morphology and motility, etc.), assessment of tubal patency, and detection of ovulation over a period of 1 year of regular unprotected sexual intercourse is normally sufficient to identify the problem. In a few cases where patients cannot be assessed, a diagnosis of unexplained infertility (UI) would be given (2). There are five factors that are commonly identified in the female reproductive system that could cause clinical infertility; (1) diminished ovarian reserve or ovulatory dysfunction (25%–30%); (2) tubal disease or blockage (20%–25%); (3) endometriosis (10%–20%); (4) uterine abnormalities such as cervical polyps, submucous uterine myoma, intrauterine adhesion, endometrial hyperplasia, and uterine malformation (0%–5%); and (5) unexplained infertility (25%–30%) (3–8). Fallopian tubes and uterine abnormalities are the main causes of female infertility, and hysteroscopy is able to diagnose the pathological factors and treat such disorders effectively (9). Furthermore, hysteroscopy can help to identify the real cause of UI. Owing to the advantages of limited trauma, high accuracy, direct visualization, and low misdiagnosis rate, hysteroscopy is the “gold standard” for diagnosing and treating macroscopic intrauterine disease (9, 10), infertility, recurrent pregnancy loss, and presurgical evaluation (11). Although the beneficial therapeutic effects of hysteroscopic surgery have been demonstrated by Longfa et al., some complications such as the formation of intrauterine adhesions post-operatively cannot be ignored (12).

Estrogen therapy is widely performed as a supplementary treatment following hysteroscopy. A previous study reports that estrogen treatment before hysteroscopy could promote blood supply, accelerate endometrial basal layer proliferation, and probe the normal endometrium during surgery (13). The latter authors discovered that preoperative estrogen can also reduce surgical times and effectively avoid re-adhesion (14–16). Most importantly, estrogen administration can normalize the menstrual cycle so that surgery can take place in the proliferative phase rather than waiting for the required phase (17, 18). Post-operatively administered estrogen can improve endometrial regeneration and menstruation, inhibit recurrent adhesions, and improve conception rate of UI patients (12, 19). However, it is still controversial whether estrogen should be given before hysteroscopy for prognosis improvement. Estrogen therapy is normally given in doses of 2 mg/day to 12 mg/day prior to surgery (12, 20–22). However, Songshu et al. believe that an oral dose of 9 mg/day can achieve the best result with minimal side effects (14). Moreover, Auclair et al. observed that continuous stimulation of large doses of estrogen without progesterone resistance could lead to endometrial lesions (23). Thus, we aimed to investigate the effect of estrogen administration before hysteroscopy on the endometrium and UI treatment. In this study, infertility patients were recruited who were subsequently treated with estrogen before hysteroscopic surgery, and the effects of estrogen on endometrial metabolism in the proliferative phase were investigated. A cutting-edge metabolomic approach was then used to identify the metabolic alterations occurring in such treatment. Using this approach, alterations in the pentose phosphate pathway (PPP) with pre-operative estrogen treatment were observed. Intervention in this PPP metabolism can reduce the risk of endometrial hyperplasia.

## Materials and Methods

### Chemicals and Reagents

Dimethyl sulfoxide (DMSO), 6-aminonicotinamide (6-AN), and 17β-estradiol were purchased from Sigma-Aldrich (St Louis, MO, USA). High-glucose Dulbecco’s Modified Eagle Medium (high-glucose DMEM), penicillin–streptomycin, trypsin-EDTA (0.25%), and fetal bovine serum were purchased from Thermo Fisher Scientific (Gibco, USA). Cell Counting Kit-8 (CCK-8) was purchased from Yeasen (China). Water (HPLC Grade), acetonitrile (HPLC Grade, 99.95%), 2-propanol (HPLC Grade, 99.9%), methanol (HPLC Grade, 99.9%), ammonium hydroxide (LC–MS grade), and acetic acid (HPLC Grade, 99.7%) were ordered from Fisher Chemical. MTBE (HPLC Grade, 99.9%) was ordered from Sigma-Aldrich. Formic acid (LC-MS Grade) was ordered from Fisher Scientific.

### Patient Recruitment

Twelve infertility patients were recruited before undergoing hysteroscopy combined with laparoscopy. Six patients were treated with estrogen (Estrogen Group) and the remainder were not (Control Group) before they all underwent hysteroscopy combined with laparoscopy. Patients in the Estrogen Group were given estradiol valerate (Progynova) at an oral dose of 2 mg daily for 7 days before hysteroscopy and this was continued for 21 days. Endometrial tissue of both estrogen-treated and control groups were collected during surgery and frozen at −80°C until metabolite extraction. The use of endometrial tissue was approved by the Obstetrics and Gynecology Hospital of Fudan University’s ethics board (the hospital’s ethics board (NO.2021-132), Shanghai, China and consent was obtained from each patient.

### Cell Culturing

The Ishikawa cells maintained in our laboratory are immortal endometrial tumor cells that can perfectly mimic the *in vitro* behavior of endometrial epithelium cells (24). Ishikawa cells are derived from a well-differentiated adenocarcinoma of human endometrial epithelium that expresses functional steroid receptors for estradiol (E2) and progesterone (P4) (25). The cell line represents an ideal model of normal endometrial epithelium cells owing to its phenotypic similarity and response to steroids, similar to physiological conditions (26). The Ishikawa cells were seeded at a density of 2,000 cells/well for 24 h until they were attached to the 96-well plate for cell proliferation measurement. For the metabolomics analysis, Ishikawa cells were seeded at a density of 2 × 10^5^ cells/well for 24 h until they were attached to the 24-well plate. After serum fasting for 24 h, the cells were treated with 100 nM estrogen or DMSO (vehicle control) for 48 h until their metabolites were extracted.

### Cell Proliferation Measurement

For the Ishikawa cells cultured in the 96-well plate, cell culture media were replaced by serum free DMEM and “fasting” for 24 h before drug treatments. Two cell proliferation assays were processed: (1) the fasted cells were treated with 5 µM of the PPP metabolism inhibitor 6-aminonicotinamide (6-AN); or 100 nM estrogen; or 100 nM estrogen along with 5 µM 6-AN; or DMSO (vehicle) for 0 h, 24 h, 48 h, 72 h, or 96 h, and subsequent cell proliferation was measured using a CCK8 kit (Yeasen); (2) the fasted cells were treated with 10 µM 6-AN; or 100 nM estrogen; or 100 nM estrogen along with 10 µM 6-AN; or DMSO (vehicle) for 0 h, 24 h, 48 h, and 72 h; cell proliferation was also measured using a CCK8 kit (Yeasen).

### Metabolite Extraction

Metabolites were extracted from endometrial tissue samples and Ishikawa cell samples following a published protocol (27). Briefly: tissue samples (2 mg) were placed in microcentrifuge tubes and smashed with stainless steel beads at 4°C for 30 min. The homogenous samples were then transferred to 15-ml centrifuge tubes and 4.5 ml ice-cold 80% methanol was added. The Ishikawa cell samples were placed in 15-ml centrifuge tubes with 4.5 ml of ice-cold 80% methanol added. The mixture (either homogenous tissue or cells) was incubated at −80°C for 2 h and centrifuged 14,000 × *g* for 10 min at 4°C. The supernatant was collected into three 1.5-ml microcentrifuge tubes while the pellets were discarded. The supernatant was dried in a SpeedVac at room temperature, and the metabolites were stored at −80°C until analyzed by UHPLC-MS.

### UHPLC-MS Metabolomic Analysis

Metabolites extracted from endometrial tissue samples and Ishikawa cells were reconstituted with 100 µl of acetonitrile:water (v:v 50:50), and 5-µl sample solutions were injected into the UHPLC-MS. The metabolites were acquired using a targeted metabolomics method that was modified from a published protocol (28). The UHPLC was equipped with an HILIC column (XBridge Amide 3.5 µm, 4.6 × 100 mm) and samples were eluted with a gradient. Two buffers, buffer A (95% water and 5% acetonitrile with 20 mM ammonium hydroxide and 20 mM ammonium acetate, pH 9.0) and buffer B (acetonitrile), were used in the gradient. The total flow of the gradient was 0.25 ml/min, which started from 0 to 0.1 min, 85% B; 3.5 min, 32% B; 12 min, 2% B; 16.5 min, 2% B; and 16–17 min, 85% B. Metabolites in the samples were acquired by a QTRAP 5500+ (AB Sciex) mass spectrometer using targeted MRM methods containing 297 transitions. All transition peaks were integrated on a MultiQuant (AB Sciex) to obtain a metabolomics peak list.

### Statistical Analyses

The metabolomics data were analyzed using MetaboAnalyst 5.0 (29) and GraphPad (Prism 8). Heatmap, PCA score plot, and VIP score were analyzed using MetaboAnalyst. Student’s *t*-test was performed to evaluate significant metabolite differences between E2 and the control group (*p < 0.05, **p < 0.01, n.s., not significant) using GraphPad (Prism 8). The metabolic pathway analysis referred to the KEGG (https://www.kegg.jp/) metabolic pathway map (30).

## Results and Discussion

Endometrial hyperplasia enlarges the glandular architecture of the human uterus and can lead to a series of unexpected consequences (31, 32) including cancerous proliferation. The overproduction of estrogen from adipose tissue in obese patients greatly contributes to endometrial hyperplasia and even endometrial cancer. For most cases of infertility treatment, oral intake of estrogen before hysteroscopy combined with laparoscopy can protect from intrauterine adhesion, as well as preparing the endometrium for embryo transplantation. There is a risk that estrogen treatment during the endometrial proliferative phase may induce side effects, including endometrial hyperplasia and endometrial cancer; therefore, it is necessary to investigate biological signaling, especially metabolomic alteration after estrogen treatment in infertility patients.

### Metabolomic Profiling of Endometrial Tissues

**Figure 1:**
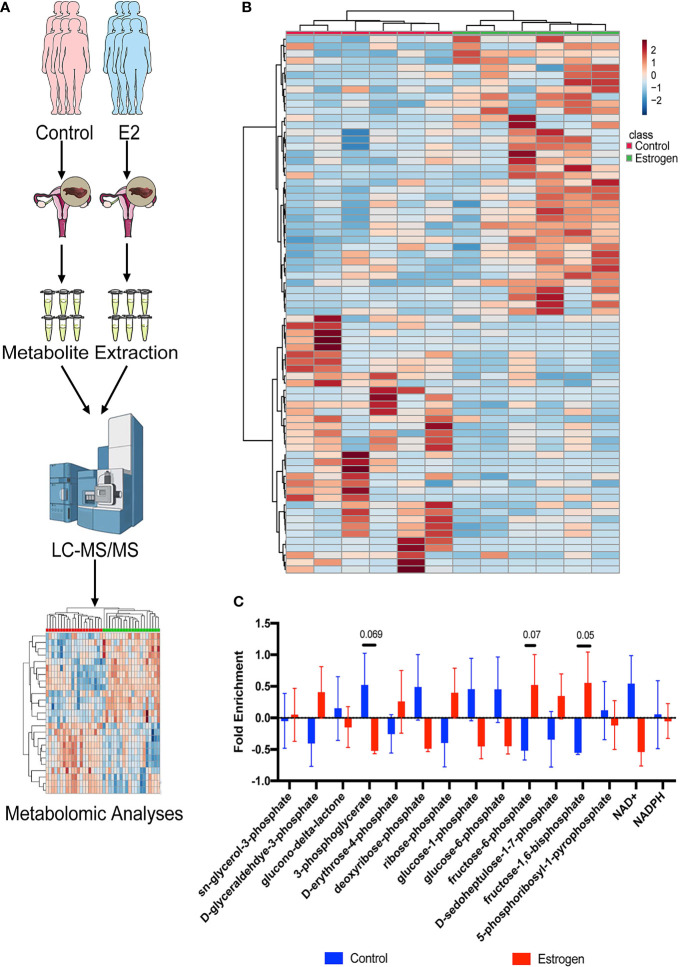
Metabolomic profiling of endometrial tissues. **(A)** Metabolomic profiling was used to analyze alterations to the metabolome in endometrial tissue of female infertility patients who were treated with estrogen. **(B)** Heat map showing metabolic changes in endometrial tissue samples. **(C)** The ratio of metabolic intermediates correlated to the PPP and glycolysis from endometrial tissue samples.

Metabolomics based on cutting-edge UHPLC-MS techniques have been widely used in the discovery of multiple clinical mechanisms and biomarkers in recent years. We used these advanced tools to analyze metabolome alterations in the endometrial tissue of infertility patients who were treated with estrogen (**Figure 1A**). We collected endometrial tissue from 12 infertility patients; half of them were treated with E2 and the remainder were not. All the tissues were processed using the same metabolite extraction procedure and injected into the UHPLC-MS. The metabolomic results indicated that estrogen can indeed affect metabolic changes in patient’s endometrial tissues (**Figure 1B**), and most of them are associated with PPP metabolism. We then checked the metabolic intermediates correlated with the PPP and glycolysis (**Figure 1C**). Both the glycolysis metabolites (pyruvate, fructose-6-phosphate, fructose-1,6-phosphate, 1,3-diphosphateglycerate, phosphoenolpyruvate, and glyceraldehyde-3-phosphate) and PPP intermediates (sedoheptulose-1,7-phosphate, erythrose-4-phosphate, and ribose-phosphate) were upregulated after estrogen treatment (**Figure 1C**). This implies that the oral intake of estrogen can affect glucose usage in infertility patients and that glucose catabolism is promoted in general. However, we did not observe an increase in glucose aerobic oxidation (**Supplemental Figure 1**). Levels of NAD+ and NADH fell in the estrogen-treated tissues; this indicated that glucose may flux to glycolysis and the PPP instead of aerobic oxidation catabolism.

### The PPP Is Promoted After Estrogen Treatment

**Figure 2:**
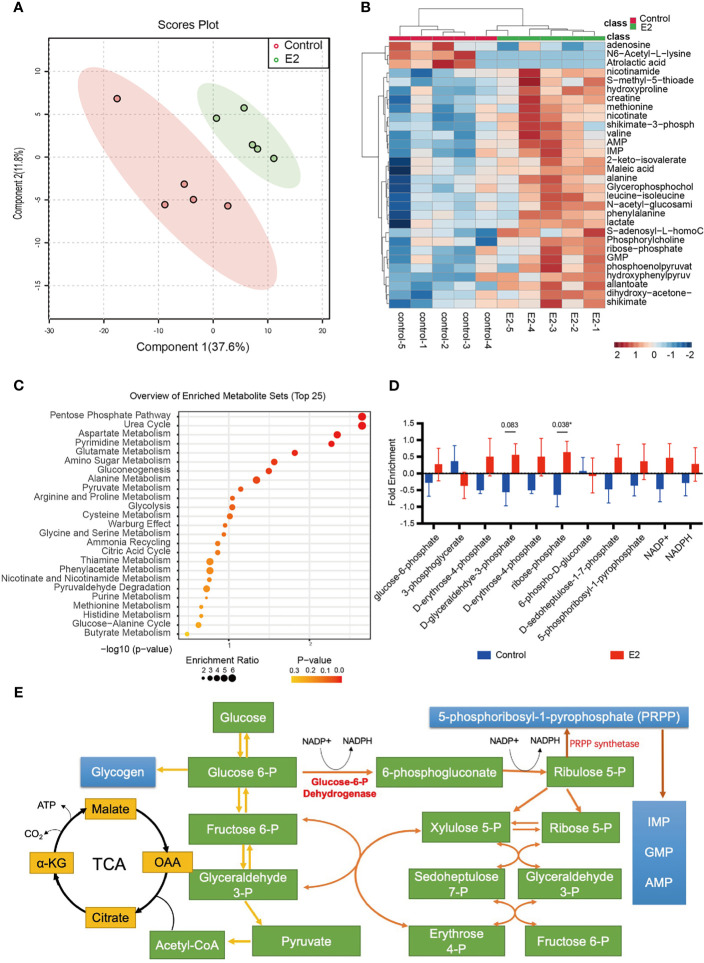
The pentose phosphate pathway is promoted after estrogen treatment. **(A)** PCA score plot of Ishikawa cells with or without estrogen treatment. **(B)** Heat map showing metabolic changes in Ishikawa cells with or without estrogen treatment. **(C)** KEGG pathway enrichment analysis of the two groups. **(D)** The ratio of metabolic intermediates correlated to the PPP and glycolysis from Ishikawa cells with or without estrogen treatment (*p-value < 0.05). **(E)** Model depicting the action of PRPP between glucose metabolism and the nucleotide synthesis.

Although we observed that glucose fluxes to glycolysis and the PPP in the endometrial tissues of infertility patients following estrogen treatment, it is still unclear if the endometrial epithelium can be directly affected by estrogen. To evaluate the effects of estrogen on endometrial epithelium cells, we treated the Ishikawa cell line with 100 nM estrogen and profiled the metabolic alterations. *In vitro* cell metabolomics results indicated that estrogen can extensively alter the metabolites of endometrial epithelium cells (**Figure 2A**). Most of the metabolites were elevated by 2 mg/day estrogen treatment (**Figure 2B**). This implies that estrogen could promote the metabolism of Ishikawa cells to generate more substrates for cell proliferation. To take a close look at the altered metabolites, we preformed metabolite set enrichment analysis (MSEA) using MetaboAnalyst 5.0 (29). The metabolite ontology results indicated that 12 metabolic pathways were significantly changed, and the top one was the PPP (**Figure 2C**), which was consistent with the findings in estrogen-treated endometrial tissue from infertility patients (**Figure 1C**). We then profiled the metabolic intermediates correlated to the PPP, which were all elevated with estrogen treatment as we expected (**Figure 2D**). It is interesting that 5-phosphoribosyl-1-pyrophosphate (PRPP) was also upregulated by estrogen. PRPP is one of the key metabolites that link glucose metabolism to nucleotide synthesis (**Figure 2E**); therefore, increased PRPP could imply that *de novo* nucleotide biosynthesis is stimulated by estrogen in endometrial epithelium cells. This result is consistent with Oliver’s discovery in 1972, who observed that the rate of purine *de novo* synthesis in an immature rat uterus was doubled at 6 h after 17β-estradiol administration (33).

### Estrogen Affects Cell Proliferation *via* PPP

**Figure 3:**
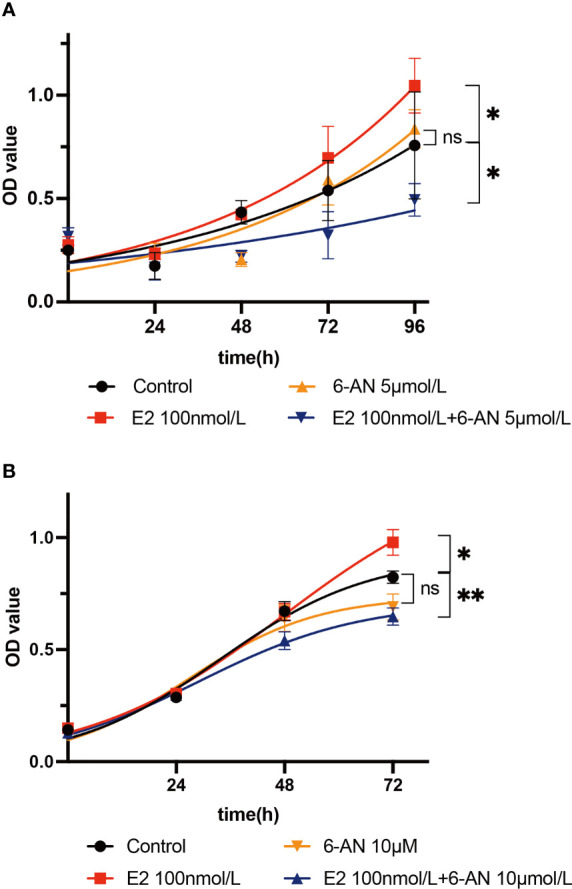
Estrogen affects cell proliferation *via* the pentose phosphate pathway. **(A)** Ishikawa cells were treated with 5 µM 6-AN; or 100 nM estrogen; or 100 nM estrogen along with 5 µM 6-AN; or DMSO for 0 h, 24 h, 48 h, 72 h, or 96 h, and cell proliferation was measured using a CCK8 kit. **(B)** Ishikawa cells were treated with 10 µM 6-AN; or 100 nM estrogen; or 100 nM estrogen along with 10 µM 6-AN; or DMSO for 0 h, 24 h, 48 h, or 72 h, and cell proliferation was measured using a CCK8 kit, *p-value < 0.05, **p-value < 0.01, n.s., not significant.

Metabolically, the *de novo* biosynthesis of nucleotides is normally linked to promote cell proliferation, especially in cancerous cells (34, 35). As we found that estrogen may affect endometrial epithelium cell glucose metabolism *via* the PPP, we set out to determine whether estrogen affects cell proliferation through such glucose catabolism. First, Ishikawa cells were treated with two different doses of 6-aminonicotinamide (5 µM and 10 µM 6-AN) and no significant cell proliferation changes were observed (**Figures 3A, B**, orange line vs. black line). 6-AN is a 6-phosphogluconate dehydrogenase inhibitor that turns glucose-6-phosphate into 6-phosphogluconate, and it inhibits glucose fluxing to PPP while it does not perturb glycolysis and aerobic respiration. Such results indicate that merely blocking the flux of glucose to the PPP is unlikely to affect endometrial epithelium cell proliferation. We then treated endometrial epithelium cells with both estrogen and 6-AN, and surprisingly noted a decelerated growth of endometrial epithelium cells to be even slower than the vehicle-treated cells, regardless of the volume of 6-AN that was added to the cell culture (**Figures 3A, B**, blue line vs. black line). The cells treated with only estrogen achieved an accelerated growth as we expected (**Figures 3A, B** red line vs. black line). Such results imply that the estrogen-promoted endometrial epithelium proliferation might be a pentose phosphate metabolism-dependent mechanism; meanwhile, turning off PPP metabolism, the endometrial epithelium cell growth would be slowed by estrogen.

### Estrogen May Stimulate PPP

It is known that estrogen can induce cell proliferation and stimulate cell growth (36–40), but there is still little evidence indicating that estrogen can alter such biological processes through metabolic pathways (41). Sun et al. showed that E2 can promote breast cancer and lymphangioleiomyomatosis tumor addiction to the PPP with upregulated G6PD enzyme activity (42). Salama et al. proposed that an E2-induced hESC proliferation would be closely linked to fluxes of glucose metabolism that upregulate aerobic glycolysis leading to such proliferation associated with PKM2 (43). Imbert-Fernandez et al. showed that E2 might also upregulate multiple glycolytic enzyme expressions and activities such as PFKFB3, resulting in increased glucose uptake and fructose 2,6-bisphosphate (F2,6BP) concentration (44). All of this evidence indicates that E2 can manipulate cellular metabolism to alter cell proliferation and other biological processes. Profiling the metabolome of estrogen-treated endometrial tissues, we believe that such hormones can alter glucose-associated metabolism in infertility patients. Glucose catabolism is one of the essential biochemical processes occurring in cells as glycolysis and the tricarboxylic acid cycle (TCA) provide energy for cell survival (**Supplemental Figure 1A**), while the PPP offers the substrates (such as nucleotides) for cell proliferation (45). As we observed that several PPP intermediates were upregulated in endometrial tissues after estrogen treatment, it can be hypothesized that estrogen stimulates the activation of pentose phosphate metabolism to promote endometrial cell proliferation. Using an endometrial epithelium immortal cell line (Ishikawa cells), we validated the promotion of cell growth function of estrogen as Ishikawa cells grew significantly faster than the vehicle-treated cells. Meanwhile, after blocking metabolism of the PPP with a chemical inhibitor (6-AN), the growth of Ishikawa cells was not affected. Surprisingly, when the endometrial epithelium cells were treated with both estrogen and 6-AN at the same time, the growth rate was slower than the vehicle controls. Such unexpected results imply that the cell growth promotion function of estrogen not only is highly dependent on activation of the PPP, but also can be regulated by the inhibition of pentose phosphate metabolism. Sun et al. proposed that the survival of estradiol-treated lymphangioleiomyomatosis xenograft mice was attenuated by the depletion of G6PD (42); Forbes et al. also observed the increased metabolic flux of the PPP in MCF-7 cells undergoing treatment with estradiol (46). Once the PPP is inhibited and glucose cannot flux to ribose and nucleotide synthesis, the estrogen treatment on endometrial epithelium diverts to preserving energy and nutrients rather than undergoing cell proliferation. Such high-dosage-induced E2-associated cell proliferation inhibition was also observed decades ago by Lewis-Wambi and Jordan in breast cancer. They found that high-dose estrogen-induced tumor regression is linked to extrinsic (Fas/FasL) and intrinsic (mitochondria) pathways (47). Srivastava et al. found that a high dosage of E2 can inhibit the differentiation of murine bone marrow monocytes and RAW 264.7 cells into mature osteoclasts (48). Researchers also found an inducible apoptosis effection of long-term high-dose E2 in cancer cells (49, 50). All this evidence implies a detrimental side to high-dosage E2 treatment.

**Figure 4:**
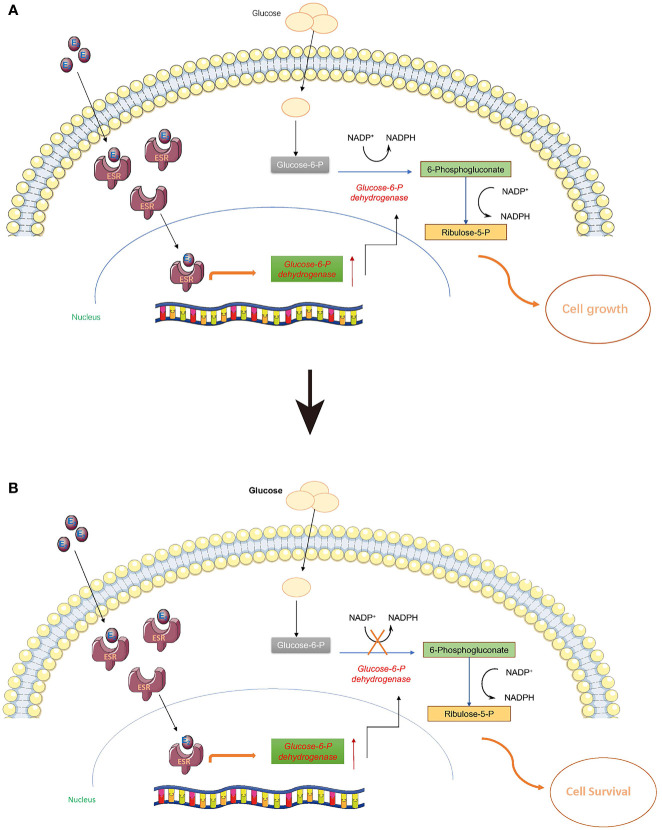
Estrogen may stimulate the pentose phosphate pathway. Estrogen can enter cells and bind to estrogen receptors (ESR) so that it can be delivered to the nucleus as a complex to stimulate a cascade biochemical reactions. **(A)** Once the pentose phosphate pathway is activated, the *de novo* biosynthesis of nucleotides can largely supply the materials (DNA and RNA) for cell proliferation. **(B)** When G6PD activity is inhibited with 6-AN, E2-ESR can promote the expression of G6PD without stimulating the *de novo* biosynthesis of nucleotides; the cells cannot proliferate, or may even reduce their growth rate to preserve sufficient energy for cell survival.

Normally, estrogen can enter cells and bind to estrogen receptors (ESR) so that it can be delivered to the nucleus as a complex to stimulate a cascade of biochemical reactions (**Figure 4**). It is widely known that ESR complexes can promote the expression of G6pd and the translation of glucose 6 phosphate dehydrogenase (G6PD) (51–53). G6PD is a critical enzyme in the conversion of glucose 6-phosphate to yield 6-phosphogluconate, and this biochemical reaction is also associated with one molecule of NADP^+^ being transformed to NADPH. Once the PPP is activated, *de novo* biosynthesis of nucleotides can largely supply the materials (deoxyribonucleic acid for DNA and ribonucleic acid for RNA) for cell proliferation (**Figure 4A**). However, when G6PD activity is inhibited with 6-AN, E2-ESR can promote the expression of G6PD without stimulating *de novo* biosynthesis of nucleotides. Cells might sense this signal of proliferation at an early time point, but without sufficient DNA and RNA, the cell cannot proliferate, or may even slow down the growth rate to preserve energy for cell survival (**Figure 4B**). Thus, we observed a decline in cell proliferation when 6-AN was added to the E2-treated cells (**Figure 3**).

In our research, the Ishikawa cells we used are cancer cells; this was because it is difficult to obtain normal cells for experiments. Although they can perfectly mimic the *in vitro* behavior of endometrial epithelium cells (24), the experimental data obtained may have certain limitations and therefore need to be further verified by systems such as organoids in future work.

## Conclusion

In this study, we recruited 12 female infertility patients to investigate metabolome alteration of endometrial epithelium treated with estrogen. Using a cutting-edge UHPLC-MS metabolomics technique, we observed that the PPP is perturbed by estrogen; it alters cell proliferation by promoting pentose phosphate pathway metabolism. We believe that such a cutting-edge metabolomics approach can lead to the discovery of pathological mechanisms in clinical infertility research.

## Data Availability Statement

The original contributions presented in the study are included in the article/**Supplementary Material**. Further inquiries can be directed to the corresponding authors.

## Author Contributions

All authors contributed to data analysis, drafting, and revision of this manuscript; gave final approval of the version to be published; and agree to be accountable for all aspects of the work.

## Funding

This work was supported by the Natural Science Foundation of Shanghai (19ZR1406800) to BL, the National Natural Science Foundation of China (NSFC 82171633), and the Natural Science Foundation of Shanghai (20ZR1408800) to CG.

## Conflict of Interest

The authors declare that the research was conducted in the absence of any commercial or financial relationships that could be construed as a potential conflict of interest.

## Publisher’s Note

All claims expressed in this article are solely those of the authors and do not necessarily represent those of their affiliated organizations, or those of the publisher, the editors and the reviewers. Any product that may be evaluated in this article, or claim that may be made by its manufacturer, is not guaranteed or endorsed by the publisher.

## References

[1] Infertility Workup for the Women’s Health Specialist: ACOG Committee Opinion, Number 781. Obstet Gynecol (2019) 133(6):e377–84. doi: 10.1097/aog.0000000000003271 31135764

[2] Gleicher N Kuhnir VA Barad DH. Unexplained Infertility. Lancet (2018) 392(10157):1516–7. doi: 10.1016/S0140-6736(18)31890-7 30496056

[3] Ray A Shah A Gudi A Homburg R. Unexplained Infertility: An Update and Review of Practice. Reprod BioMed Online (2012) 24(6):591–602. doi: 10.1016/j.rbmo.2012.02.021 22503948

[4] Takasaki A Tamura I Okada-Hayashi M Orita T Tanabe M Maruyama S. Usefulness of Intermittent Clomiphene Citrate Treatment for Women With Polycystic Ovarian Syndrome That Is Resistant to Standard Clomiphene Citrate Treatment. Reprod Med Biol (2018) 17(4):454–8. doi: 10.1002/rmb2.12219 PMC6194245 30377399

[5] Smith S Pfeifer SM Collins JA. Diagnosis and Management of Female Infertility. JAMA (2003) 290(13):1767–70. doi: 10.1001/jama.290.13.1767 14519712

[6] Evers JL. Female Subfertility. Lancet (2002) 360(9327):151–9. doi: 10.1016/S0140-6736(02)09417-5 12126838

[7] Grimbizis GF Camus M Tarlatzis BC Bontis JN Devroey P. Clinical Implications of Uterine Malformations and Hysteroscopic Treatment Results. Hum Reprod Update (2001) 7(2):161–74. doi: 10.1093/humupd/7.2.161 11284660

[8] National Collaborating Centre for Ws, Children’s H. National Institute for Health and Clinical Excellence: Guidance. In: Fertility: Assessment and Treatment for People with Fertility Problems. London: Royal College of Obstetricians & Gynaecologists Copyright © 2013 (2013). National Collaborating Centre for Women’s and Children’s Health. 25340218

[9] Parry JP Isaacson KB. Hysteroscopy and Why Macroscopic Uterine Factors Matter for Fertility. Fertil Steril (2019) 112(2):203–10. doi: 10.1016/j.fertnstert.2019.06.031 31352959

[10] Bettocchi S Ceci O Vicino M Marello F Impedovo L Selvaggi L. Diagnostic Inadequacy of Dilatation and Curettage. Fertil Steril (2001) 75(4):803–5. doi: 10.1016/s0015-0282(00)01792-1 11287038

[11] Lindsay TJ Vitrikas KR. Evaluation and Treatment of Infertility. Am Fam Physician (2015) 91(5):308–14. 25822387

[12] Kou L Jiang X Xiao S Zhao YZ Yao Q Chen R. Therapeutic Options and Drug Delivery Strategies for the Prevention of Intrauterine Adhesions. J Control Release (2020) 318:25–37. doi: 10.1016/j.jconrel.2019.12.007 31830539

[13] Song D Xia E Xiao Y Li TC Huang X Liu Y. Management of False Passage Created During Hysteroscopic Adhesiolysis for Asherman’s Syndrome. J Obstet Gynaecol (2016) 36(1):87–92. doi: 10.3109/01443615.2015.1030601 26207696

[14] Liu AZ Zhao HG Gao Y Liu M Guo BZ. Effectiveness of Estrogen Treatment Before Transcervical Resection of Adhesions on Moderate and Severe Uterine Adhesion Patients. Gynecol Endocrinol (2016) 32(9):737–40. doi: 10.3109/09513590.2016.1160375 26982384

[15] Zhang L Wang M Zhang Q Zhao W Yang B Shang H. Estrogen Therapy Before Hysteroscopic Adhesiolysis Improves the Fertility Outcome in Patients With Intrauterine Adhesions. Arch Gynecol Obstet (2019) 300(4):933–9. doi: 10.1007/s00404-019-05249-y 31350664

[16] Shargaieva O Kuske L Rappich J Unger E Nickel NH. Building Blocks of Hybrid Perovskites: A Photoluminescence Study of Lead-Iodide Solution Species. Chemphyschem (2020) 21(20):2327–33. doi: 10.1002/cphc.202000479 PMC7702157 32786129

[17] Myers EM Hurst BS. Comprehensive Management of Severe Asherman Syndrome and Amenorrhea. Fertil Steril (2012) 97(1):160–4. doi: 10.1016/j.fertnstert.2011.10.036 22100167

[18] Chang Y Duan H Shen X Wang S Guo Z Chen S. Controversy in the Management of Oestrogen Therapy Before Hysteroscopic Adhesiolysis: A Systematic Review and Meta-Analysis. Reprod BioMed Online (2020) 41(4):715–23. doi: 10.1016/j.rbmo.2020.06.012 32782169

[19] Tonguc EA Var T Yilmaz N Batioglu S. Intrauterine Device or Estrogen Treatment After Hysteroscopic Uterine Septum Resection. Int J Gynaecol Obstet (2010) 109(3):226–9. doi: 10.1016/j.ijgo.2009.12.015 20152976

[20] Zhou Q Wu X Dai X Yuan R Qi H. The Different Dosages of Estrogen Affect Endometrial Fibrosis and Receptivity, But Not SDF-1/CXCR4 Axis in the Treatment of Intrauterine Adhesions. Gynecol Endocrinol (2018) 34(1):49–55. doi: 10.1080/09513590.2017.1328050 28531361

[21] Guo J Li TC Liu Y Xia E Xiao Y Zhou F. A Prospective, Randomized, Controlled Trial Comparing Two Doses of Oestrogen Therapy After Hysteroscopic Adhesiolysis to Prevent Intrauterine Adhesion Recurrence. Reprod BioMed Online (2017) 35(5):555–61. doi: 10.1016/j.rbmo.2017.07.011.Citedin:Pubmed 28784336

[22] Xiao S Wan Y Xue M Zeng X Xiao F Xu D. Etiology, Treatment, and Reproductive Prognosis of Women With Moderate-to-Severe Intrauterine Adhesions. Int J Gynaecol Obstet (2014) 125(2):121–4. doi: 10.1016/j.ijgo.2013.10.026.Citedin:Pubmed 24598346

[23] Auclair MH Yong PJ Salvador S Thurston J Colgan TTJ Sebastianelli A. Guideline No. 392-Classification and Management of Endometrial Hyperplasia. J Obstet Gynaecol Can (2019) 41(12):1789–800. doi: 10.1016/j.jogc.2019.03.025 31785798

[24] Nishida M Kasahara K Kaneko M Iwasaki H Hayashi K. Establishment of a New Human Endometrial Adenocarcinoma Cell Line, Ishikawa Cells, Containing Estrogen and Progesterone Receptors. Nihon Sanka Fujinka Gakkai Zasshi (1985) 37(7):1103–11. 4031568

[25] Tamm-Rosenstein K Simm J Suhorutshenko M Salumets A Metsis M. Changes in the Transcriptome of the Human Endometrial Ishikawa Cancer Cell Line Induced by Estrogen, Progesterone, Tamoxifen, and Mifepristone (RU486) as Detected by RNA-Sequencing. PloS One (2013) 8(7):e68907. doi: 10.1371/journal.pone.0068907 23874806 PMC3712916

[26] Bilgic E Guzel E Kose S Aydin MC Karaismailoglu E Akar I. Endocannabinoids Modulate Apoptosis in Endometriosis and Adenomyosis. Acta Histochem (2017) 119(5):523–32. doi: 10.1016/j.acthis.2017.05.005 28549792

[27] Huang H Yuan M Seitzer P Ludwigsen S Asara JM. Isosearch: An Untargeted and Unbiased Metabolite and Lipid Isotopomer Tracing Strategy From HR-LC-MS/MS Datasets. Methods Protoc (2020) 3(3):54. doi: 10.3390/mps3030054 PMC7563207 32751454

[28] Yuan M Breitkopf SB Yang X Asara JM. A Positive/Negative Ion-Switching, Targeted Mass Spectrometry-Based Metabolomics Platform for Bodily Fluids, Cells, and Fresh and Fixed Tissue. Nat Protoc (2012) 7(5):872–81. doi: 10.1038/nprot.2012.024 PMC3685491 22498707

[29] Pang Z Chong J Zhou G de Lima Morais DA Chang L Barrette M. Metaboanalyst 5.0: Narrowing the Gap Between Raw Spectra and Functional Insights. Nucleic Acids Res (2021) 49(W1):W388–96. doi: 10.1093/nar/gkab382 PMC8265181 34019663

[30] Kanehisa M Furumichi M Tanabe M Sato Y Morishima K. KEGG: New Perspectives on Genomes, Pathways, Diseases and Drugs. Nucleic Acids Res (2017) 45(D1):D353–61. doi: 10.1093/nar/gkw1092 PMC5210567 27899662

[31] Montgomery BE Daum GS Dunton CJ. Endometrial Hyperplasia: A Review. Obstet Gynecol Surv (2004) 59(5):368–78. doi: 10.1097/00006254-200405000-00025 15097798

[32] Armstrong AJ Hurd WW Elguero S Barker NM Zanotti KM. Diagnosis and Management of Endometrial Hyperplasia. J Minim Invasive Gynecol (2012) 19(5):562–71. doi: 10.1016/j.jmig.2012.05.009 22863972

[33] Oliver JM. A Possible Role for 5-Phosphoribosyl 1-Pyrophosphate in the Stimulation of Uterine Purine Nucleotide Synthesis in Response to Oestradiol-17. Biochem J (1972) 128(4):771–7. doi: 10.1042/bj1280771 PMC1173897 4344697

[34] Ben-Sahra I Howell JJ Asara JM Manning BD. Stimulation of *De Novo* Pyrimidine Synthesis by Growth Signaling Through Mtor and S6K1. Science (2013) 339(6125):1323–8. doi: 10.1126/science.1228792 PMC3753690 23429703

[35] Ricoult SJ Yecies JL Ben-Sahra I Manning BD. Oncogenic PI3K and K-Ras Stimulate *De Novo* Lipid Synthesis Through mTORC1 and SREBP. Oncogene (2016) 35(10):1250–60. doi: 10.1038/onc.2015.179 PMC4666838 26028026

[36] Spady TJ McComb RD Shull JD. Estrogen Action in the Regulation of Cell Proliferation, Cell Survival, and Tumorigenesis in the Rat Anterior Pituitary Gland. Endocrine (1999) 11(3):217–33. doi: 10.1385/ENDO:11:3:217 10786818

[37] Liang J Shang Y. Estrogen and Cancer. Annu Rev Physiol (2013) 75:225–40. doi: 10.1146/annurev-physiol-030212-183708 23043248

[38] Yager JD Davidson NE. Estrogen Carcinogenesis in Breast Cancer. N Engl J Med (2006) 354(3):270–82. doi: 10.1056/NEJMra050776 16421368

[39] Bogush TA Dudko EA Beme AA Bogush EA Kim AI Polotsky BE. Estrogen Receptors, Antiestrogens, and non-Small Cell Lung Cancer. Biochemistry (Mosc) (2010) 75(12):1421–7. doi: 10.1134/s0006297910120011 21314611

[40] Kon OL. Estrogens, Antiestrogens and Cell Proliferation. Bioessays (1989) 10(6):210–4. doi: 10.1002/bies.950100608 2662968

[41] Kulkoyluoglu-Cotul E Arca A Madak-Erdogan Z. Crosstalk Between Estrogen Signaling and Breast Cancer Metabolism. Trends Endocrinol Metab (2019) 30(1):25–38. doi: 10.1016/j.tem.2018.10.006 30471920

[42] Sun Y Gu X Zhang E Park MA Pereira AM Wang S. Estradiol Promotes Pentose Phosphate Pathway Addiction and Cell Survival *via* Reactivation of Akt in mTORC1 Hyperactive Cells. Cell Death Dis (2014) 5:e1231. doi: 10.1038/cddis.2014.204 24832603 PMC4047866

[43] Salama SA Mohammad MA Diaz-Arrastia CR Kamel MW Kilic GS Ndofor BT. Estradiol-17beta Upregulates Pyruvate Kinase M2 Expression to Coactivate Estrogen Receptor-Alpha and to Integrate Metabolic Reprogramming With the Mitogenic Response in Endometrial Cells. J Clin Endocrinol Metab (2014) 99(10):3790–9. doi: 10.1210/jc.2013-2639 24471565

[44] Imbert-Fernandez Y Clem BF O’Neal J Kerr DA Spaulding R Lanceta L. Estradiol Stimulates Glucose Metabolism *via* 6-Phosphofructo-2-Kinase (PFKFB3). J Biol Chem (2014) 289(13):9440–8. doi: 10.1074/jbc.M113.529990 PMC3979387 24515104

[45] Patra KC Hay N. The Pentose Phosphate Pathway and Cancer. Trends Biochem Sci (2014) 39(8):347–54. doi: 10.1016/j.tibs.2014.06.005 PMC4329227 25037503

[46] Forbes NS Meadows AL Clark DS Blanch HW. Estradiol Stimulates the Biosynthetic Pathways of Breast Cancer Cells: Detection by Metabolic Flux Analysis. Metab Eng (2006) 8(6):639–52. doi: 10.1016/j.ymben.2006.06.005 16904360

[47] Lewis-Wambi JS Jordan VC. Estrogen Regulation of Apoptosis: How Can One Hormone Stimulate and Inhibit? Breast Cancer Res (2009) 11(3):206. doi: 10.1186/bcr2255 19519952 PMC2716493

[48] Srivastava S Toraldo G Weitzmann MN Cenci S Ross FP Pacifici R. Estrogen Decreases Osteoclast Formation by Down-Regulating Receptor Activator of NF-Kappa B Ligand (RANKL)-Induced JNK Activation. J Biol Chem (2001) 276(12):8836–40. doi: 10.1074/jbc.M010764200 11121427

[49] Yao K Lee ES Bentrem DJ England G Schafer JI O’Regan RM. Antitumor Action of Physiological Estradiol on Tamoxifen-Stimulated Breast Tumors Grown in Athymic Mice. Clin Cancer Res (2000) 6(5):2028–36. 10815929

[50] Zhang Y Zhao H Asztalos S Chisamore M Sitabkhan Y Tonetti DA. Estradiol-Induced Regression in T47D:A18/Pkcalpha Tumors Requires the Estrogen Receptor and Interaction With the Extracellular Matrix. Mol Cancer Res (2009) 7(4):498–510. doi: 10.1158/1541-7786.Mcr-08-0415 19372579 PMC2743931

[51] Gordon MN Mobbs CV Finch CE. Pituitary and Hypothalamic Glucose-6-Phosphate Dehydrogenase: Effects of Estradiol and Age in C57BL/6J Mice. Endocrinology (1988) 122(2):726–33. doi: 10.1210/endo-122-2-726 3338416

[52] Ibim SE Randall R Han P Musey PI. Modulation of Hepatic Glucose-6-Phosphate Dehydrogenase Activity in Male and Female Rats by Estrogen. Life Sci (1989) 45(17):1559–65. doi: 10.1016/0024-3205(89)90422-0 2586221

[53] Park S Safi R Liu X Baldi R Liu W Liu J. Inhibition of Erralpha Prevents Mitochondrial Pyruvate Uptake Exposing NADPH-Generating Pathways as Targetable Vulnerabilities in Breast Cancer. Cell Rep (2019) 27(12):3587–3601.e4. doi: 10.1016/j.celrep.2019.05.066 31216477 PMC6604861

