# Supplementary material for: Metabolomics Analysis Discovers Estrogen Altering Cell Proliferation via the Pentose Phosphate Pathway in Infertility Patient Endometria
Source: Front Endocrinol (Lausanne). 2021 Nov 15;12:791174. doi: 10.3389/fendo.2021.791174 (PMC8636142; doi:10.3389/fendo.2021.791174)
Supplement: Supplementary file 1 [file DataSheet_1.docx]

Supplementary Material

# Supplementary Figures and Tables


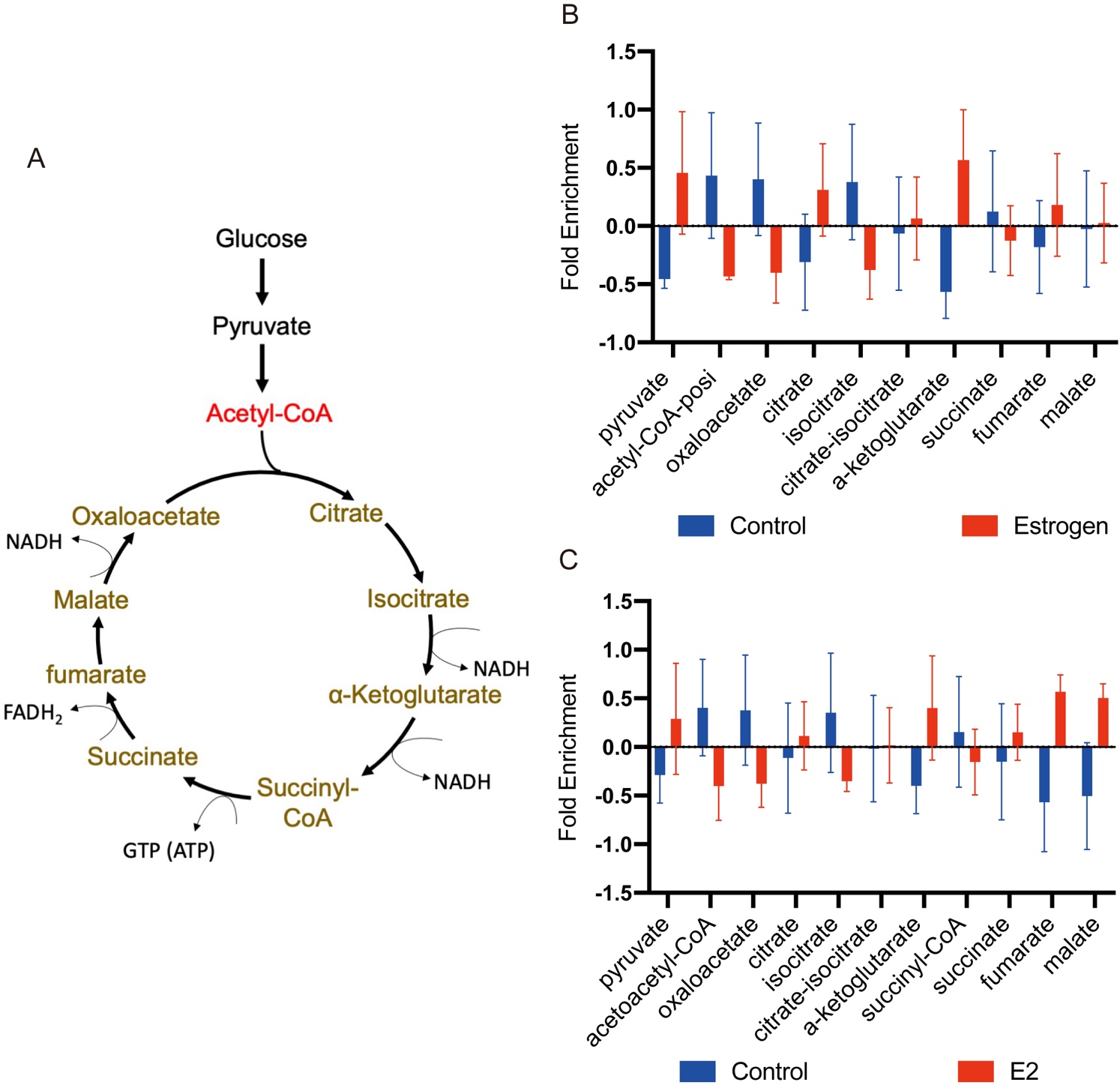


**Supplemental Figure 1. Glucose aerobic oxidation did not increase after estrogen treatment**

**(A)** Model depicting of glucose aerobic oxidation. **(B)** The ratio of metabolic intermediates correlated to tricarboxylic acid cycle (TCA) from endometrial tissue samples; **(C)** The ratio of metabolic intermediates correlated to TCA from Ishikawa cell with or without estrogen treatment.
